# A Case Report of Kratom-Induced Psychiatric Decompensation

**DOI:** 10.1192/j.eurpsy.2022.2139

**Published:** 2022-09-01

**Authors:** E. Garrels, G. Gill, S. Mitra, P. Korenis

**Affiliations:** BronxCare Health System, Psychiatry, Bronx, United States of America

**Keywords:** Kratom, intellectual disability, Addiction, Psychosis

## Abstract

**Introduction:**

Kratom (*Mitragyna speciosa*) is an herb found in South East Asia belonging to the Rubiacea family, the active constituents being Mitragynine and 7-hydroxymitragynine. Sold as a dietary supplement in the form of a leaf, tablet, and powder, it has been gaining popularity as a natural supplement to alleviate pain, anxiety, depression, and manage opioid withdrawal symptoms. Our case report centers around a patient encountered with high-dose Kratom use who presented to our Psychiatric ER with psychosis.

**Objectives:**

The objectives of this case report are to raise awareness regarding the use of a newly popular substance easily available over-the-counter and the potential impacts it has on mental health.

**Methods:**

PubMed was searched for the criteria Kratom AND Intellectual Disability, with a secodary search for Kratom AND Psychosis.

**Results:**

A 29-year-old male with a past psychiatric history of Schizoaffective Disorder, Borderline Intellectual Functioning, Polysubstance Use, ADHD, and six prior suicide attempts was brought to the Psychiatric ED by ambulance activated by his mother for severe psychiatric decompensation following ingestion of 270 pills of Kratom over the course of three days.

**Conclusions:**

This case report further increases awareness of the dangers of Kratom use as well as brings to light the psychoactive properties of Kratom. This case report exposes areas where research can further expand understanding regarding the impacts Kratom can have on psychiatric populations.

**Disclosure:**

No significant relationships.

